# Genomics Enabled Breeding Strategies for Major Biotic Stresses in Pea (*Pisum sativum* L.)

**DOI:** 10.3389/fpls.2022.861191

**Published:** 2022-05-18

**Authors:** Ashok Kumar Parihar, Jitendra Kumar, Debjyoti Sen Gupta, Amrit Lamichaney, Satheesh Naik SJ, Anil K. Singh, Girish P. Dixit, Sanjeev Gupta, Faruk Toklu

**Affiliations:** ^1^Crop Improvement Division, ICAR-Indian Institute of Pulses Research (ICAR-IIPR), Kanpur, India; ^2^All India Coordinated Research Project on Chickpea, ICAR-IIPR, Kanpur, India; ^3^Indian Council of Agricultural Research, New Delhi, India; ^4^Department of Field Crops, Faculty of Agricultural, Cukurova University, Adana, Turkey

**Keywords:** biotic stresses, genomics, proteomics, marker assisted breeding, speed breeding

## Abstract

Pea (*Pisum sativum* L.) is one of the most important and productive cool season pulse crops grown throughout the world. Biotic stresses are the crucial constraints in harnessing the potential productivity of pea and warrant dedicated research and developmental efforts to utilize omics resources and advanced breeding techniques to assist rapid and timely development of high-yielding multiple stress-tolerant–resistant varieties. Recently, the pea researcher’s community has made notable achievements in conventional and molecular breeding to accelerate its genetic gain. Several quantitative trait loci (QTLs) or markers associated with genes controlling resistance for fusarium wilt, fusarium root rot, powdery mildew, ascochyta blight, rust, common root rot, broomrape, pea enation, and pea seed borne mosaic virus are available for the marker-assisted breeding. The advanced genomic tools such as the availability of comprehensive genetic maps and linked reliable DNA markers hold great promise toward the introgression of resistance genes from different sources to speed up the genetic gain in pea. This review provides a brief account of the achievements made in the recent past regarding genetic and genomic resources’ development, inheritance of genes controlling various biotic stress responses and genes controlling pathogenesis in disease causing organisms, genes/QTLs mapping, and transcriptomic and proteomic advances. Moreover, the emerging new breeding approaches such as transgenics, genome editing, genomic selection, epigenetic breeding, and speed breeding hold great promise to transform pea breeding. Overall, the judicious amalgamation of conventional and modern omics-enabled breeding strategies will augment the genetic gain and could hasten the development of biotic stress-resistant cultivars to sustain pea production under changing climate. The present review encompasses at one platform the research accomplishment made so far in pea improvement with respect to major biotic stresses and the way forward to enhance pea productivity through advanced genomic tools and technologies.

## Introduction

Pea (*Pisum sativum* L.), being cultivated throughout the world, either for food, fodder, and feed, is considered an important winter season food legume (Rubiales et al., 2019; Parihar et al., 2020). Cotyledons’ color of pea grains varies from yellow, green, and orange that are used in the human diet in different forms such as *dal, stew, chhola, vegetables, snacks, soup, chat*, and flour, while whole seeds are mainly used as animal feed (Mahajan et al., 2018; Singh et al., 2018). Nutritionally, pea seeds are considered to have about 21–33% protein and 56–74% carbohydrate, with an average iron, selenium, zinc, and molybdenum of about 97, 42, 41, and 12 ppm, respectively (Parihar et al., 2016, 2021). Therefore, it serves as an important ingredient in providing nutritional security for resources poor people in developing countries. Moreover, its consumption minimizes the risk of several chronic diseases such as diabetes (Marinangeli and Jones, 2011), subsides blood cholesterol levels (Ekvall et al., 2006), improves cardiovascular health (Singh et al., 2013), possesses cancer prevention attributes (Kalt, 2001; Steer, 2006), administers body weight, and improves gastrointestinal affairs (Fernando et al., 2010; Lunde et al., 2011).

It is being cultivated widely across many countries in the world (Parihar et al., 2021). Its worldwide cultivated area has increased from 6.58 to 8.09 mha and production from 10.44 to 16.21 mt since 2010. Canada, Russia, China, India, and the United States are the major pea-producing countries (Parihar et al., 2020); however, the United States shares the highest total production of pea (39.33%), followed by Europe (36.98%) and Asia (18.09%). At present, its average productivity is about 2.0 t/ha globally, which recorded an increase of about 36% in a decade (2007–2017), but the potential productivity of this crop is up to 5.0 t/ha in several countries including Netherland, Denmark, Belgium, Germany, and Finland harvests about 3.45–5.01 t/ha (Toker and Mutlu, 2011). However, countries such as India, China, Australia, and Myanmar are recording very low productivity of less than 2.00 t/ha (FAO, 2021). During the past few decades, the gain in yield of pea (15.3 kg/ha/year) is relatively low as compared to other crops, which could be majorly attributed to the least investment in the pea research program (Rubiales et al., 2019). Also, the susceptibility of a pea toward many abiotic/biotic stress is another reason for low productivity which becomes a serious threat to its sustainable productivity especially under changing climatic conditions (Parihar et al., 2020). The most devastating diseases that affect the productivity of pea are powdery mildew (PM), ascochyta blight (AB), rust (PR), wilt (FW), and root rots (Parihar et al., 2013; Mahajan et al., 2018), of which PM caused by *Erysiphe* pisi (DC.), *E. baeumleri* (Magnus) (U. Braun & S. Takam.), and *E. trifolii* (Grev.) has the potential of reducing seed yield by 25–80% (Warkentin et al., 1996; Ghafoor and McPhee, 2012). PR caused by *Uromyces viciae-fabae* (Pers.) J. Schröt. *or U. pisi* (Pers.) de Bary is reported to cause yield losses up to 30% (Barilli et al., 2010, 2018; Singh et al., 2015) while, AB, results due to a mixture of fungal species [*Ascochyta pisi* (Lib.), *Peyronellaea pinodes* (Berk. & A. Bloxam), *Phoma medicaginis* var. *pinodella* (L.K. Jones), *P. Koolunga* (Davidson), and *P. glomerata* (Corda) (Wollenw. & Hochapfel)], is one of the most complex and severe diseases worldwide (Bretag et al., 2006; Tran et al., 2014) with a potential of reducing grain yield by about 60% (Liu et al., 2016). Fusarium root rot (FRR) incited by *Fusarium solani* f. sp. *pisi* (W.C. Snyder & H.N. Hansen), which may occur in both dry and wet field conditions, reduces yield significantly (Porter, 2010). Similarly, fusarium wilt (FW) caused by *F. oxysporum* f. sp. *pisi* (W.C. Snyder & H.N. Hansen) has about 11 different races (Gupta and Gupta, 2019), of which races 1 and 2 are distributed widely affecting the productivity of pea significantly, whereas races 5 and 6 are sporadically distributed (Infantino et al., 2006; Bani et al., 2018). A disease caused by *Aphanomyces euteiches* (Drechsler) is common root rot (CRR) and is prevalent in the United States, Europe, and Canada causes wilting of the roots (Wicker et al., 2003; Pilet Nayel et al., 2005; Chatterton et al., 2015; Desgroux et al., 2016; Wu et al., 2018). Several insect pests such as pod borer complex [*Helicoverpa armigera* (Hübner), *Etiella zinckenella* (Treitschke), and *Polyommatus boeticus* L.], bruchid (*Bruchus pisorum* L.) pea leaf weevil (*Sitona lineatus* L.), leaf miners [*Chromatomyia horticola* (Goureau)], stem fly [*Melanagromyza phaseoli* (Vanschuytbroeck)], aphids [*Acyrthospihon pisum* (Harris)], and cut worms [*Agrotis ipsilon* (Hufnagel)] seriously reduce the yield of pea by affecting the crop growth (Sharma, 2000; Yadav and Patel, 2015; Yadav et al., 2019). Pod damage of about 40% has been observed in pea due to pod borer complex infestation (Dahiya and Naresh, 1993).

The development of resistant cultivars to the biotic and abiotic stresses is an outstanding tactic to enhance the productivity of any crop including pea. Therefore, knowledge of the genetics of disease and pest resistance is essentially required to breed the resistant/tolerant cultivars. In addition to this, genomic advances especially the accessibility of draft genome sequence of pea (Kreplak et al., 2019) have facilitated the identification of the genes responsible for disease and pest resistance/tolerance and also helped in uncovering the genetics of quantitatively inherited resistance of several major diseases and pests. Moreover, genomics has also facilitated modernizing the conventional breeding for rapid and precise development of resistant cultivars in crop plants including pea. Information on genetics, genomics, and breeding of biotic stress resistance in pea is scattered and only limited attempts were made to review the different aspects of biotic stress resistance (Fondevilla and Rubiales, 2012; Smýkal et al., 2012; Rubiales et al., 2015; Tayeh et al., 2015a). Recently, Mahajan et al. (2018) discussed the genetic improvement in pea in relation to biotic stresses; however, the information provided was largely related to legumes in general and in brief about pea. Thus, an effort is made through this review to make available the comprehensive information pertaining to genetic and genomic advancement at one platform as well as to share a futuristic road map using modern genomic and genetic tools in pea breeding that could aid the crop breeders in developing high-yielding multiple stress resilient pea cultivars.

## Current Status of Genetic Resources

**FIGURE 1:**
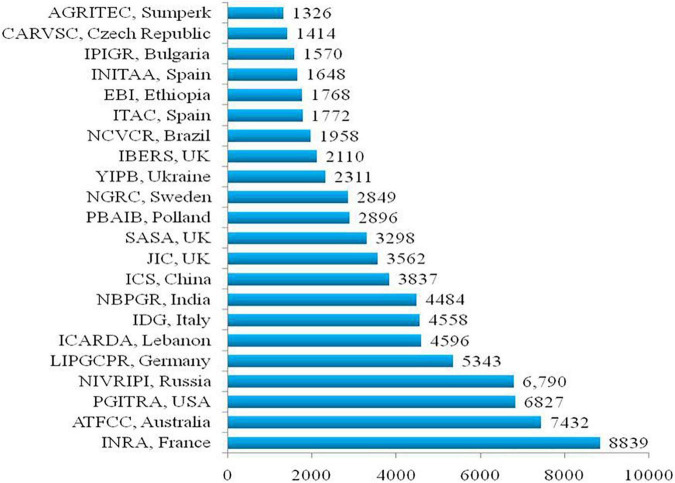
Major pea germplasm holding organizations worldwide (Warkentin et al., 2015; https://www.genesys-pgr.org/).

Genetic improvement in a target crop species requires availability and judicious exploitation of genetic resources. Globally, more than 98,000 pea accessions, comprised of advanced breeding lines (13%), landraces (38%), mutant stocks (5%), wild species (2.6%), and cultivars (34%), are available and conserved in diverse genebanks (Smýkal et al., 2015; Warkentin et al., 2015; Rubiales et al., 2019; Coyne et al., 2020). The National Institute for Agricultural Research (INRA), France, Australian Grains Genebank (AGG), N.I. Vavilov Research Institute of Plant Industry, Russia, US Department of Agriculture (USDA), United States, Leibniz Institute of Plant Genetics and Crop Plant Research, Gatersleben, Germany, and International Center for Agricultural Research in the Dry Areas (ICARDA), Lebanon are the six leading active pea germplasm repositories in the world with about 8,839, 7,432, 6,790, 6,827, 5,343, and 4,596 accessions, respectively (Figure 1). The National Germplasm Repositories of various countries also hold a good number of pea accessions such as 4,558 accessions in Italy, 3,837 in China, 4,484 in India, 3,298 in the United Kingdom, 2,896 in Poland, 2,849 in Sweden, 2,311 in Ukraine, and 2,110 in Aberystwyth University, United Kingdom. Besides, seven other countries hold > 1,000 accessions of *Pisum* in their national germplasm treasury (Figure 1). Interestingly, the National Genebank of Israel possesses a collection of crop wild relatives (CWRs) such as *Pisum fulvum* and *P. sativum* subsp. *elatius* var. *pumilio*, which contributes to about 2% of the entire preserved germplasm (Smýkal et al., 2013, 2015; Warkentin et al., 2015). This share of CWR has accessions to *P. fulvum* (706), *P. s*. subsp. *elatius* (624), *P. s*. subsp. *sativum* (syn. *P. humile/syriacum*; 1562), and *P. abyssinicum* (540) (Smýkal et al., 2013). Besides CWR and cultivated accessions, 575 and 122 accessions of pea mutant stocks are also available at the John Innes Collection, the United Kingdom and the Institute of Plant Genetics Resources Collection, Bulgaria, respectively (Smýkal et al., 2015). A Targeted Induced Local Lesions in Genomes (TILLING) population of 9,000 lines (Coyne et al., 2020) and fast neutron generated deletion mutant resources (around 3,000 lines) are also available, which are being exploited to identify various developmental genes (Smýkal et al., 2015). Internationally, several web-portals have been developed using the database of pea genetic resources such as the European Cooperative Program on Plant Genetic Resources, Cool Season Food Legume Database, Genetic Resources Information Network and System-wide Information Network for Genetic Resources, and KnowPulse for keeping records and disseminating the information related to pea genetic resources.

Crop wild relatives that include *Pisum* species and subspecies are in general a source of countless fascinating traits including various yield attributing parameters (Mikić et al., 2013). Besides, it is a source of resistance to several biotic stresses, e.g., pea seed weevil (Clement et al., 2002, 2009), PM (Fondevilla et al., 2007b; Esen et al., 2019), PR (Barilli et al., 2010), AB (Jha et al., 2012), and broomrape (Fondevilla et al., 2005). The significance of CWR has been demonstrated by successfully introducing a novel dominant gene (*Er3)*, responsible for resistance to *E. pisi* from *P. fulvum* (Sharma and Yadav, 2003; Fondevilla et al., 2008a). Moreover, some *P. fulvum* accessions were reported to show resistance against bruchid, broomrape, and *Mycosphaerella pinodes* and are subsequently being utilized in hybridization programs (Fondevilla et al., 2005; Coyne et al., 2020). Similarly, resistance to PR (Barilli et al., 2010, 2018) and AB (Fondevilla et al., 2005; Jha et al., 2012) has been observed in *P. fulvum*. Diversity for the *eIF4E* gene and novel alleles for virus resistance has also been identified from CWR (Ashby et al., 2011; Konečná et al., 2014). In a recent report, the relationship between neoplasm and pea weevil (*Bruchus pisorum* L.) damage was not established in F_1_ and F_2_ derived from the inter-subspecific crosses of *P. sativum* subsp. *sativum* (with neoplasm) and *P. sativum* subsp. *elatius* (without neoplasm) in field conditions (Sari et al., 2020).

Interestingly, the germplasm with the least commercial acceptance in terms of colored seed coat and flowers was accredited as a wonderful resistance source for root rot diseases (Grunwald et al., 2003; Weeden and Porter, 2007) and *Aphanomyces* (Hamon et al., 2011). Most significantly, the resistance to different biotic stresses can also be transferred from *Lathyrus species* that are harbored in the tertiary pea gene pool (Patto et al., 2007, 2009), preferably through the utilization of contemporary biotechnological techniques. Most recently, super-early progeny derived from an interspecific cross between *P. sativum* and *P. fulvum* flowered in 13–17 days and set pod in 18–29 days after emergence. Such progeny could be used as a complementary to “speed breeding,” to generate more than six generations per year in an appropriate climate compartment (Sari et al., 2021). Significant contributions have been made toward the identification of resistant genetic resources for major biotic stresses in pea (Table 1), which might be utilized in breeding programs and further genetic analysis for the identification of new resistance genes.

**TABLE 1:** Potential resistance source of different biotic stresses in field pea.

Biotic stress	Germplasm/variety/wild relatives	Country	References
Powdery mildew	9057, 9370, 9375, 10609, 10612, 18293, 18412, 19598, 19611, 19616, 19727, 19750, 19782, 20126, 20152, 20171, It-96, No. 267, and No. 380	Pakistan	Azmat et al., 2012
	Medora, PS9910188, PS810765, PS810324, Stirling, PS0010128, PS8 10240, PS710048, PS810191, 3272, 3273, Lifter, Franklin, and Fallon	Pakistan	Nisar et al., 2006
	*P. fulvum* (P660-4)	Spain	Fondevilla et al., 2007b
	HFP4, EC598878, EC598538, EC598757, EC598704, EC598729, EC598535, EC598655, EC598816, EC381866, IC278261, IC267142, IC218988, IC208378, IC208366	India	Rana et al., 2013
	LE 25, ATC 823, KPMR-10, T-10, P-185,6533, 6587, 6588, JI 210, DMR 4, DMR 7, DMR 20	India	Ghafoor and McPhee, 2012
	HFP 9907 B, Pant Pea -42, VL Matar 42, IPFD 99-13, IPFD 1-10, IPF 99-25, Pusa prabhat, Ambika	India	Dixit and Gautam, 2015
	Highlight, AC Tamor, Tara, Mexique 4, Stratagem, JI 210, JI 1951, JI 1210, JI 2480	Canada	Tiwari et al., 1997
	Glenroy, Kiley, Mukta, M257-3-6, M257-5-1, PSI 11, ATC 1181	Australia	Liu et al., 2003
	GPHA-9, GPHA-19	Ethiopia	Assen, 2020
	JI2480	India	Katoch et al., 2010
Rust	IPF-2014-16, KPMR-936 and IPF-2014-13,	India	Das et al., 2019a
	PJ 207508, C 12, Wisconsin, DMR 3, Pant P 5, Pant P 8, Pant 9, HFP 8711 and HUDP 15, IPFD 1-10	India	Chaudhary and Naimuddin, 2000; Dixit and Gautam, 2015
	JP-4, FC-1, Pant P 11, HUDP 16, JPBB-3, HUP 14	India	Dhall, 2015
Downey mildew	Mukta, Snowpeak	Australia	Davidson et al., 2004
Pea seed-borne mosaic virus (PSbMV)	PI 193586, PI 193835	Ethiopia	Hagedorn and Gritton, 1973
*Pseudomonas syringae* pv. *pisi* (race 6, 8)	JI0130	Spain	Martín-Sanz et al., 2012
*Pseudomonas syringae* pv. *Pisi* (race 8)	Forrimax, JI2546, PI-277852, ZP1328, Cherokee, Corallo, Lincoln, JI2385, PM29, PM232, PM33, JI1829, ZP1282, ZP0104, ZP1301, ZP0123, ZP0168	Spain	Martín-Sanz et al., 2012
Mycosphaerella blight (*Mycosphaerella pinodes)*	CN 112432, CN 112441, CN 112513	Canada	Jha et al., 2012
	*P. fulvum* (P651), Radley	Spain	Fondevilla et al., 2005
Stem fly (*Melanagromyza phaseoli*)	P-4039, P-4107	India	Vishal and Ram, 2005
Leaf miner (*Chromatomyia horticola*)	P-4107	India	Vishal and Ram, 2005
Pea weevil (*Bruchus pisorum*)	*P. fulvum* (ATC113)	Australia	Hardie et al., 1995; Byrne et al., 2008
Pulse beetle (*Callosobruchus chinensis* L.)	*P*. *sativum* (ACP 11), *P*. *elatius* (AWP 442) *P*. *fulvum* (AWP 600, AWP 601)	Turkey	Esen et al., 2019
Fusarium root rot (*Fusarium solani* f. sp. *pisi)*	PI215766, PI244121	United States	Grunwald et al., 2003
	JI 1794 (*P. sativum* subsp. *elatius*).	United States	Hance et al., 2004
	PI125839, PI125840, PI175226, PI220174, PI223526, PI223527, PI226561 and PI227258	United States	Porter, 2010
*Fusarium oxysporum f.* sp. *pisi*	JI1412, JI1760 (*P. sativum* ssp.), P633 (*P. sativum* ssp. *arvense*), P42 (*P. sativum* ssp. *elatius*)	Spain	Bani et al., 2012, 2018

## Current Knowledge on Genetics for Disease Resistance

Knowledge of genes controlling disease resistance is important to accelerate the success of any breeding program (Shashikumar et al., 2010). Understanding gene action/effects operating in a particular breeding population helps to select a suitable parent for hybridization and breeding procedure for making genetic improvements of resistance against that disease (Sharma et al., 2013). Notably, the pea is acknowledged as the original model organism and was utilized in the finding of Mendel’s laws of inheritance, which laid the foundation for modern plant genetics. In the recent years, inheritance has been studied for resistance attributes of disease in pea by several researchers (Lamprecht, 1948; Yarnell, 1962; Blixt, 1974; Gritton, 1980; Kalloo and Bergh, 1993; Kumar et al., 2006; Amin et al., 2010), and genes were identified and mapped using conventional gene mapping approaches. Varieties with inbuilt resistance are the most appropriate, competent, and economic strategies for tackling biotic stresses. Therefore, comprehensive efforts have been made to understand the inheritance of biotic stresses. Inheritance study for PM revealed that it is being operated by two recessive genes (*er1* and *er2*) and one dominant gene (*Er3*) (Fondevilla et al., 2007a). A recent report illustrated that PM resistance is operated *via er1* owing to the non-functioning of gene *PsMLO1* (Humphry et al., 2011). The gene *er2* is reported to provide complete resistance to PM but is efficient only in location-specific breeding (Tiwari et al., 1997; Fondevilla et al., 2006), while gene *Er3* confers resistance in *P. fulvum* (Fondevilla et al., 2007a,^2010^).

With regard to PR resistance, it was reported to be operated by a single dominant gene (*Ruf*) (Tyagi and Srivastava, 1999); however, the polygenic nature of gene action (Singh and Ram, 2001) and partial dominance of a single gene in conjunction with minor and additive genes (2–3) (Singh et al., 2012) have also been found recently. A single dominant gene governs resistance toward races 1 and 2 of *F. oxysporum* f. *pisi*, pea enation mosaic virus, *F. solani* f. sp. *pisi*, brown root rot, bacterial blight, downy mildew, and other root rot diseases of pea, whereas a recessive gene regulates resistance to pea seed borne mosaic virus (*sbm*), yellow bean mosaic virus (*mo*), pea mosaic virus (*pmv*), and bean virus (Amin et al., 2010; Mohan et al., 2013). However, Davidson et al. (2004) reported downy mildew to be controlled by a single dominant gene and two complementary recessive genes. The nature of inheritance of AB and FRR resistance has been reported to be regulated by many genes (Kraft, 1992; Fondevilla et al., 2007b; Carrillo et al., 2014b; Jha et al., 2017). The pod resistance for pea weevil is quantitatively controlled whereas the seed resistance is operated by three (*pwr1*, *pwr2*, and *pwr3*) major recessive alleles (Byrne et al., 2008). The neoplasm appearance on pods is controlled by a single dominant gene and its expressivity is influenced by one or a combination of environmental factors (Sari et al., 2020).

## Exploitation of Genetic Knowledge Through Traditional Breeding Approaches for Biotic Stress Resistance

Numerous biotic stresses including FW, AB, PM, PR, FRR, and CRR are serious threats to pea production (Bohra et al., 2014). These diseases are reported to occur in a severe form in almost all the pea growing countries. Therefore, efforts have been made to exploit the available genetic knowledge of resistance through conventional breeding for these key biotic stresses for developing resistant cultivars (Fondevilla and Rubiales, 2012; Ghafoor and McPhee, 2012). To develop high yielding pea cultivars possessing PM resistance, three genes, namely, *er1*, *er2*, and *Er3* have been exploited successfully using conventional breeding approaches (Heringa et al., 1969; Fondevilla et al., 2007c). The *er1* gene has the highest existence in resistant pea accessions followed by the *er2* gene, which is harbored in restricted accessions (Tiwari et al., 1997). Therefore, the *er1* gene that provides resistance through the pre-penetration resistance mechanism has been largely exploited in most pea improvement programs worldwide (Fondevilla et al., 2006). PR is another serious disease, scattered across the countries where the pea is being cultivated. Resistance to PR has been reported to be polygenic (Singh et al., 2012) and oligogenic (Vijayalakshmi et al., 2005). AB or black spot disease is one of the most devastating diseases of peas causing yield setbacks of up to 60% (Xue et al., 1996; Liu et al., 2016). Being seed borne, the rate of transmission from seed to sapling for *A. pisi* and *P. pinodes* is 40–100% (Maude, 1966; Xue, 2000), with an ability to remain viable on seeds for 5–7 years (Wallen, 1955). To date, the absolute resistant source for AB has not been identified; however, a prominent scale of resistance was found in accession (P651) of *P. fulvum*, which is being actively utilized in pea improvement (Wroth, 1998; Sindhu et al., 2014). The polygenic inheritance pattern of AB makes the development of resistant cultivars through conventional breeding very difficult. The FRR is considered a serious bottleneck in harnessing the full potential of a cultivar (Bisby, 1918; Jones, 1923). The condensed soil with a temperature of 18–24°C is the ideal thermal regime for the proliferation of FRR (Kraft and Boge, 2001). Unfortunately, complete resistance to this disease is yet to be explored; however, genetic sources carrying partial tolerance to this disease are available in pea (Gretenkort and Helsper, 1993; Porter, 2010). Noteworthy, the majority of the colored flower accessions portrayed a good level of resistance to FRR as compared to white colored flower accessions (Grunwald et al., 2003). Also, the polygenic inheritance of this disease has made the development of resistant varieties more complicated (Muehlbauer and Kraft, 1973; Kraft, 1992). FW is another severe production menace scattered around the world caused by *Fusarium oxysporum*. f. sp. *pisi* and causes absolute yield loss under appropriate environmental circumstances (Aslam et al., 2019). The most favorable soil temperature for FW disease development is 23–27°C. In total, 11 different races of fusarium have been discovered considering its virulence (Gupta and Gupta, 2019); of them, races 1 and 2 have become cosmopolitan; on the contrary, races 5 and 6 are prevailing in some areas (Bani et al., 2018). Among these races, race 1 is considered the most devastating and dominating (Kraft and Pfleger, 2001). Being a soil-borne pathogen, it may outlast for a prolonged period below the ground without pea crop (Gupta and Gupta, 2019). McPhee et al. (1999) recognized resistance sources against races 1 and 2 and used them to breed resistant cultivars. Interestingly, one CWR accession (PI 344012) having resistance to races 1 and 2 has been identified. Knowledge of inheritance is vital for incorporating any attribute of interest in the targeted genotype. Therefore, the inheritance pattern of resistance to *Fop* races 1, 5, and 6 have been studied and confirmed that it is monogenic with dominance in nature, while resistance to race 2 is regulated quantitatively (McPhee et al., 1999, 2012; Rispail and Rubiales, 2014; Bani et al., 2018). The monogenic dominant resistance is successfully introgressed in many pea cultivars (McPhee, 2003). The integration of quantitatively operated resistance in a targeted background is a cumbersome task wherein molecular markers can support significantly to accelerate the introgression process. For such traits, visual selection always remains long-lasting and labor exhaustive. Thus, modern genomic tools and techniques have paved a way for questing, utilizing, and choosing the naturally available sources of resistance against FW in pea (McClendon et al., 2002; Smýkal et al., 2012).

In pea under congruent circumstances particularly under excess moisture in the soil, CRR reduces grain yield significantly by severe damage to the root framework and subsequent wilting of the infected plant (Wu et al., 2018). Unfortunately, the existing old school disease management approaches such as crop rotation and seed treatments are incapable of controlling this disease completely, owing to the prolonged persistence of the pathogen in the form of oospores, which can contaminate crops at any phase. Consequently, resistant cultivar development has been advocated as an ultimate aim in the pea breeding scheme. Few accessions of pea having moderate resistance to CRR have been identified and subsequently used in breeding programs for developing cultivars (Pilet Nayel et al., 2002, 2005; Roux-Duparque et al., 2004; Moussart et al., 2007; Pilet Nayel et al., 2007; Hamon et al., 2011; McGee et al., 2012; Conner et al., 2013; Hamon et al., 2013; Lavaud et al., 2015). However, polygenic inheritance of this disease and its linkage with some objectionable attributes such as lengthy internodes, anthocyanin content, and delayed-flowering made it difficult to breed CRR-tolerant cultivars (Marx et al., 1972; Pilet Nayel et al., 2002).

## Toward Genomic-Based Disease and Insect-Pest Resistance Breeding

### Mapping Gene/Quantitative Trait Loci Using Molecular Markers

Traditional gene mapping could not be used widely to map the genes/quantitative trait loci (QTLs) regulating disease resistance because of narrow variability and their polygenic inheritance pattern. Moreover, quantitatively inherited traits are highly influenced by environmental conditions; therefore, the DNA-based markers are widely exploited to map genes/QTLs regulating quantitatively inherited traits in pea. In this crop, DNA-based markers that include STMS (Haghnazari et al., 2005); ISSR (Lázaro and Aguinagalde, 2006), SRAP (Esposito et al., 2007), SNP (Duarte et al., 2014), IRAP (Smýkal et al., 2008a), RBIP (Smýkal et al., 2008b), EST-SSR (Teshome et al., 2015), and SSR (Handerson et al., 2014; Negisho et al., 2017; Mohamed et al., 2019) have been developed and successfully utilized to compute genetic variations. However, similar to other crop species, only SSR makers have become popular owing to their low cost, rapidness, polymorphism, and reliable (Snowdon and Friedt, 2004). More recently, next-generation sequencing has authorized the quick discovery of SNPs and the development of an array for genotyping in pea (Leonforte et al., 2013; Duarte et al., 2014; Sindhu et al., 2014). The initial linkage maps were developed in pea utilizing various molecular markers, which were further used in mapping genes/QTLs controlling biotic stress tolerance. The genes such as *er 1, er2, and Er3* and their alleles conferring resistance to PM have been mapped using different types of markers (Table 2). In pea, sequencing of cDNA belonging to *PsMLO1* has identified a new allele *er1-6* of gene *er1* that has been validated by a closely linked specific SSR marker (Sun et al., 2016). In addition to this, alleles, namely, *er1-8* and *er1-9* have been mapped using co-dominant functional markers and validated in pea (Sun et al., 2019). The single dominant gene controlling FW resistance has also been mapped using dominant and co-dominant markers (Jiang, 2013), which were not appropriate for marker-assisted selection (MAS) due to their poor linkage with gene and dominant nature. Thus, Jain et al. (2015) recently designed a co-dominant CAPS marker with 94% accuracy and found that it was helpful in the selection of resistance toward *F. oxysporum* race 1. QTL mapping has been followed for genes regulating partial or intricate inherited resistance and recognized major or minor QTLs for biotic stress tolerance in pea. For example, molecular mapping has identified one major gene (*Ruf*)/QTL (*Up1*, *Qruf)* and one minor QTL (*Qruf1*) for PR resistance (Vijayalakshmi et al., 2005; Barilli et al., 2010; Rai et al., 2011). However, markers associated with these genes/QTLs were not close enough (>5.0-cm distance) for utilization in MAS. Further validation of markers linked with QTL *Qruf* and *Qruf1* did not show complete discrimination between PR susceptible and resistant genotypes limiting their application for marker-assisted breeding (MAB) (Singh et al., 2015). However, high-density molecular maps based on SNP makers and the use of isogenic lines (NILs) and heterogeneous inbred family (HIF) populations have provided opportunities for fine mapping of the genes/QTLs and identified more closely linked makers for precise MAS (Mohan et al., 1997; Tuinstra et al., 1997). The SNP marker-mediated linkage mapping has identified three QTLs (*UpDSII, UpDSIV, and UpDSIV.2*) for PR resistance (Barilli et al., 2018). For AB resistance, various QTL mapping studies have recognized various genomic regions concerned with the regulation of resistance (Table 3; Timmerman-Vaughan et al., 2002; Taran et al., 2003; Fondevilla et al., 2008b). Recently, Jha et al. (2015) have identified SNPs within the linked genes, namely, *RGA-G3A* (RGA-G3Ap103) and *PsDof1* (PsDof1p308), which displayed a noteworthy relationship with AB resistance. Correspondingly in another report association of nine QTLs with resistance to AB has been reported in an interspecific population derived by crossing *P. sativum* (Alfetta) and *P. fulvum* (P651), of which, only QTLs *abIII-1* and *abI-IV-2* were found to be stable over the locations/years (Jha et al., 2016), which were further fine mapped in HIF populations (Jha et al., 2017). Furthermore, selective genotyping was done utilizing genotyping-by-sequencing (GBS) in RILs recognizing eight novel SNP markers within the abI-IV-2 QTL with no extra SNPs in the QTL abIII-1. Similarly, several QTLs explaining phenotypic variation up to 53.4% for polygenic inherited FRR resistance have been recognized using SSR and SNP markers (Coyne et al., 2019). The genome-wide association study (GWAS) refined or validated the previously reported QTLs and identified new loci for resistance to *A. euteiches* (Desgroux et al., 2016), which identified 52 QTLs including six previously identified QTLs for its resistance. However, Desgroux et al. (2018) employed a comparative GWAS approach for resistance to *A. euteiches* in a large set of contrasting pea genotypes (266) using 14,157 SNP markers and identified 11 genomic intervals having significant association with resistance to *A. euteiches* and also confirmed numerous QTLs reported previously. One SNP marker, mapped to the major QTL *Ae-Ps7.6*, was linked with disease resistance and root system architecture, which can be employed in regular pea breeding programs to reduce root rot incidence in pea.

**TABLE 2:** Available genetic maps for different biotic stresses in field pea (*Pisum sativum* L.).

S. No.	Population	Population size	Type of population	Markers	Marker type	Total map distance (cM)	References
**Powdery mildew (*Erysiphe pisi*)**							
1	Kaspa × Yarrum	106	RIL	821	SSR and SNPs	1910	Sudheesh et al., 2014
2	Kaspa × ps1771	106	RIL	852	SSR and SNPs	1545	Sudheesh et al., 2014
3	C2 × Messire	100	F2	720	RAPD/SCAR	–	Fondevilla et al., 2008a
4	Slow × JI1794	51	RIL	200	RAPD/RFLP	–	Timmerman et al., 1994
5	Almota × 88V1.11	111	F2	200	RAPD/RFLP	–	Timmerman et al., 1994
6	Lincoln/JI2480	111	F2	152	SSR	51.9	Katoch et al., 2010
7	Radley × Highlight	99	F2:3	416	RAPD/SCAR	–	Tiwari et al., 1998
8	PG 3*^HFP 4^* × PG 3	208	F2	633	RAPD/SCAR	–	Srivastava et al., 2012
9	Majoret × 955180	192	F2	315	SSR	49.9	Ek et al., 2005
10	Solara × Frilene-derived mutant	230	F2	585	ISSR, RAPDs, AFLPs	66.4	Pereira et al., 2010
11	Sparkle × Mexique	–	F2	–	RAPD/SCAR	–	Tonguç and Weeden, 2010
12	Bawan 6 × DDR-11	102	F2	9	SCAR/SSR	–	Sun et al., 2016
13	WSU 28 × G0004389	120	F2:3	20	SCAR/SSR	–	Sun et al., 2019
14	Bawan 6 × G0004400	119	F2:3	20	SCAR/SSR	–	Sun et al., 2019
15	G0001778 × Bawan 6	71	F2:3	5	SSR	–	Sun et al., 2016
16	Qizhen 76 × Xucai 1	91	F2	148	SSR	–	Sun et al., 2015
17	Xucai 1 × Bawan 6	161	F2	148	SSR	–	Sun et al., 2015
**Rust (*Uromyces pisi, Uromyces fabae)***							
1	IFPI3260 × IFPI3251	94	F3	146	RAPDs and STSs	1283.3	Barilli et al., 2010
2	HUVP 1 × FC 1	136	RIL	153	SSRs, RAPD, and STSs	634	Rai et al., 2011
3	IFPI3260 × IFPI3251	84	RIL	12,058	DArT, SNP, SSR, and STS	1877.45	Barilli et al., 2018
**Ascochyta blight (*Mycosphaerella pinodes*)**							
	JI1089 × JI296	–	–	–	–	–	Clulow et al., 1991
1	Erygel × 661	174	F2	62	RFLP, RAPD	550	Dirlewanger et al., 1994
2	A88 × Rovar	133	RIL	96	RFLP, RAPD, and AFLP	1050	Timmerman-Vaughan et al., 2002
3	Carneval × MP1401	88	RILs	239	AFLPs, RAPDs, and STSs	1274	Taran et al., 2003
4	A26 × Rovar and A88 × Rovar	148	F2	99	RAPDs, RFLPs, AFLPs, and STSs	930	Timmerman-Vaughan et al., 2004
5	JI296 × DP	135	RIL	206	RAPD, SSR and STS	1061	Prioul et al., 2004
6	P665 × Messire	111	RIL	303	SSRs	1188.97	Fondevilla et al., 2008b
7	P665 × Messire	111	RIL	248	SSRs	1119.46	Fondevilla et al., 2011; Carrillo et al., 2014a,b
8	Alfetta × P651	51	RIL	10,985	SNPs (GBS)	86.3	Jha et al., 2017
9	Carerra × CDC Striker	134	RIL	3389	SNPs	1008.8	Gali et al., 2018
**Fusarium root rot (*Fusarium solani f. sp. pisi)***							
1	Carman × Reward	71	RIL	213	Microsatellite marker (SSRs)	53.1	Feng et al., 2011
2	DSP (W6 17516) × 90–2131 (PI 557501)	111	RIL	10 gene based markers	CAPS and dCAPS	1323	Coyne et al., 2015
3	Baccara × PI 180693	178	RILs	914	SNPs	1073	Coyne et al., 2019
4	JI1794 × Slow	51	RILs	–	–	1289	Timmerman-Vaughan et al., 1996; Hance et al., 2004
5	Afghanistan”(*sym2*) × A1078-239	19		–	–	–	Weeden and Porter, 2007
6	CMG × PI220174	225	RILs	–	–	–	Weeden and Porter, 2007
**Fusarium wilt (*Fusarium oxysporum*. f. sp. *pisi)***							
1	K586 × Torsdag	139	RILs	355	RAPD	1139	Laucou et al., 1998
2	“Lifter”/“Radley” Shawnee”/“Bohatyr	393, 187	RILs	13	CAPS, SSR	–	Jain et al., 2015
3	Shawnee × Bohatyr	187	RILs	272	RAPDs and SSRs	1716	McPhee et al., 2012
4	Green Arrow × PI 179449	80	RILs	72	TRAP	–	Kwon et al., 2013
**Common root rot (*Aphanomyces euteiches*)**							
1	Puget × 90–2079	127	RILs	324	AFLPs, RAPDs, SSRs, ISSRs, STSs, isozymes	1094	Pilet Nayel et al., 2002; Loridon et al., 2005; Hamon et al., 2013
2	Puget × 90–2079	127	RILs	324	AFLPs and RAPDs	1523	Pilet Nayel et al., 2005
3	Baccara × PI180693, Baccara × 552	356	RILS	224	SSRs, RAPD and RGA	1652	Hamon et al., 2011
	Baccara × PI180693	178	RIL	4620	SNPs	705.2	Hamon et al., 2011, 2013; Duarte et al., 2014; Tayeh et al., 2015a
4	DSP × 90–2131	111	RILs	168	RAPDs, RFLPs and SSRs	1046	Hamon et al., 2013
5	Pea-Aphanomyces collection	175		13,204	SNPs	NA	Desgroux et al., 2016
6	Pea accessions	266		14,157	SNPs	NA	Desgroux et al., 2018
7	MN313 × OSU1026	45		–	–	–	Weeden et al., 2000
**Pseudomonas (*Pseudomonas syringae pv. pisi*)**							
1	JI15 × JI399	77	RILs	151	RFLPs	1700	Ellis et al., 1992
2	Vinco × Hurst’sGreenshaft, Partridge × EarlyOnward	–	–	–	–	–	Hunter et al., 2001
3	JI281 × JI399	53	RILs	421	RFLPs	2300	Hall et al., 1997
4	P665 × Messire	111	RILs	248	RAPD, STSs, SSR, and EST	1188.58	Fondevilla et al., 2012
**Broomrape (*Orobanche crenata*)**							
1	P665 × Messire	115	F2	217	RAPD and STS	1770	Valderrama et al., 2004
2	P665 × Messire	111	RILs	246	RAPDs, STSs, ESTs	1214	Fondevilla et al., 2010
**Pea weevil (*Bruchus pisorum)***							
1	Pennant × ATC113	270	RILs	155	SSRs	2686	Aryamanesh et al., 2014
2	P665 × Messire	108	RILs	6540	SNPs (DArTseq platform)	2503	Aznar-Fernández et al., 2020
**Aphid (*Acyrthosiphon pisum)***							
1	*P. fulvum* IFPI3260 × *P. fulvum* IFPI3251	84	–	12,058	DArT, SNP, SSR and STS	1877.45	Barilli et al., 2020
**Pea seed-borne mosaic virus (PSbMV)**							
1	88V1.11 × 425	88	F2	–	RFLP, RAPD, allozyme	–	Timmerman et al., 1993

**TABLE 3:** Genomic region or markers associated with resistance to different biotic stresses in field pea (*Pisum sativum* L.).

Trait	Marker name and type	Gene/QTLs	Distance (cM)	Linkage group	References
Fusarium root rot (*Fusarium solani* f. sp. *Pisi*)	AA416/SSR, AB60/SSR	*QTL*	NA	VII	Feng et al., 2011
	CAPS/dCAPS	*Fsp-Ps2.1, Fsp-Ps6.1, Fsp-Ps3.1, Fsp-4.1, Fsp-Ps7.1*	8.9–28.5	IIa, IIIb, VI, VII	Coyne et al., 2015
	Ps900203/SNP, Ps900299/SNP	*Fsp-Ps 2.1, Fsp-Ps3.2, Fsp-Ps3.3*	23.5–49.3	II, III	Coyne et al., 2019
Rust (*Uromyces fabae*)	SC10-82_360_/RAPD, SCRI- 71_1000_/RAPD	*Ruf*	10.8–24.5	–	Vijayalakshmi et al., 2005
	AA446/SSR, AA505/SSR, AD146/SSR, AA416/SSR	*Qruf, Qruf1*	7.3–10.8	VII	Rai et al., 2011
	AA121/SSR, AD147/SSR	*Qruf2*	6.0	I	Rai et al., 2016
Rust (*U. pisi*)	OPY11_1316_/RAPD, OPV17_1078_/RAPD	*Up1*	6–13.4	III	Barilli et al., 2010
	AD280/SSR, 3567800/DArT, 3563695/DArT, 3569323/DArT,	*UpDSII, UpDSIV, UpDSIV.2*	1.5–5.0	II, IV	Barilli et al., 2018
Fusarium wilt (*Fusarium oxysporum*. f. sp. *Pisi)*, race1	p254/RFLP	*Fw*	6.0	IV	Dirlewanger et al., 1994
	ACG :CAT_222/AFLP ACC :CTG_159/AFLP, Y15_1050/RAPD/	*Fw*	1.4–4.6	III	McClendon et al., 2002
	Y15_*999/*SCAR	*Fw*	–	III	Okubara et al., 2005
	AD134_213/SSR, AA5_225/SSR, AA5 _235/SSR, AB111_166/SSR, AD73/SSR, AB30/SSR AD85_178/SSR	*Fw*	2.5–12.3	III	Loridon et al., 2005
	*Fw_Trap_480/SCAR*, *Fw_Trap_340/SCAR*, *Fw_Trap_220/SCAR*	*Fw*	1.2	III	Kwon et al., 2013
	Aux1.SNP1, Hlhrep_SNP6, Hlhrep_SNP1, Cwi1_SNP3, Cwi1.SNP1, PPT2.SNP1, FVE.SNP6, PM34like.SNP2, ProteasB.SNP2, PFK_SNP1, Subt_SNP2, Sus3_SNP8, Trans_SNP1, TE002G22_SNP3	*Fw*	–	I, II, III, V, VI, VII	Cheng et al., 2015
	THO/CAPS, AnMtL6, Mt5_56, PR X1TRAP13, TC112650/SSR, TC112533/SSR	*Fw*	0.5–3.9	III	Jain et al., 2015
Fusarium wilt, race 2	PSMPSAD171/SSR	*Fnw*	–	–	McPhee et al., 2004
	AC22_185/SSR, AD171_197/SSR, AB70_203/SSR, AD180_161/SSR, AB85-284	*Fnw 4.1, Fnw 3.1, Fnw 3.2*	–	3, 4	McPhee et al., 2012
Fusarium wilt, Race5	U693a/RAPD, T3_650/RAPD	*Fwf*	5.6–5.8	II	Okubara et al., 2002
	*Aatp*	*Fwf*	9.1	II	Coyne et al., 2000
Powdery mildew	p236/RFLP	*er-1*	9.8	VI	Dirlewanger et al., 1994
	OPD10_650_/RAPD	*er-1*	2.1	VI	Timmerman et al., 1994
	*ScOPD-10_650/_SCAR*	*er-1*	3.7	VI	Rakshit, 1997
	OPL-6_1900_/RAPD, Sc-OPE-16_1600_/RAPD	*er-1*	2–4	VI	Tiwari et al., 1998
	Sc-OPO-18_1200_/RAPD	*er-1*	0.0	VI	Tiwari et al., 1998
	*ScOPD-10_650/_SCAR*	*er-1*	3.4	VI	Janila and Sharma, 2004
	OPO-02_1400_/RAPD, OPU-17_1000_/RAPD	*er-1*	4.5–10.3	VI	Janila and Sharma, 2004
	*PSMPSAD60/SSR*, *PSMPSAA374/SSR*, *PSMPA5/SSR, PSMAD51/SSR*	*er-1*	10.4–14.9	VI	Ek et al., 2005; Loridon et al., 2005
	SCW4_637_, SCAB_1874_	*Er-3*	2.8	IV	Fondevilla et al., 2008a
	OPW04_637/RAPD, OPC04_640/RAPD, OPF14_1103/RAPD, OPAH06_539/RAPD, OPAG05_1240/RAPD, OPAB01_874,	*Er-3*	0.0–6.3	IV	Fondevilla et al., 2008a
	BA9/RAPD, Act2B/RAPD, OD15/RAPD, BC210/RAPD, BC483/RAPD, OB11/RAPD, BC407/RAPD	*er-1*	8.2	VI	Tonguç and Weeden, 2010
	OPX17_1400/ScX17_1400	*er-2*	2.6	III	Katoch et al., 2010
	OPO06_1100y_/SCAR, OPT06_480_/SCAR and AGG/CAA_125_/SCAR, OPE161600/SCAR and A5420y/SSR	*er-1*	0.5–23.0	VI	Pereira et al., 2010
	OPB18/RAPD	*er-1*	11.2	VI	Nisar and Ghafoor, 2011
	OPB18_430_	*er-1*	11.2	VI	Nisar and Ghafoor, 2011
	GIM-300/SmlI/CAPS	*er1-5*	–	VI	Pavan et al., 2011
	ScOPX04_880_/SCAR, ScOPD-10_650_*/SCAR*	*er-1*	0.6–2.8	VI	Srivastava et al., 2012
	*er1-1*/AsuHPI-B/CAPS, *er1-4*/AgsI/CAPS, *er1-2*/MGB/STS, *er1-3*/XbaI/dCAPS, *er1-5*/HRM54/HRM	*er1-1, er1-4, er1-2, er1-3, er1-5*	–	VI	Pavan et al., 2013
	c5DNAmet; PSMPSAD60	*er-1*	8.1–15.4	VI	Sun et al., 2015
	AD60/SSR, c5DNAmet	*er-1*	8.1–15.4	VI	Sun et al., 2015
	c5DNAmet; PSMPSAD60	*er-1*	9.0–11.9	VI	Wang et al., 2015
	ScOPD10-_650/SCAR_, ScOPE16-_1600/SCAR_, PSMPSAD60/SSR, PSMPSA5/SSR, c5DNAmet,	*er-1*	4.2–26.2	VI	Sun et al., 2016
	InDel111–120	*er-1-7*	4.2	VI	Sun et al., 2016
	SNP1121/SNP	*er1-6*		VI	Sun et al., 2016
	AD60/SSR; c5DNAmet/SSR	*er1-6*	8.8–22.8	VI	Sun et al., 2016
	*KASPar-er1-1, KASPar-er1-3, KASPar-er1-4, KASPar-er1-5, KASPar-er1-6, KASPar-er1-7, KASPar-er1-10, KASPar-er1-11*	*er-1*	–	VI	Ma et al., 2017
	c5DNAmet, AA200/SSR, PSMPSAD51/SSR, OPX04-880/SSR,	*er-1*	3.5–12.2	VI	Sun et al., 2019
	KASPar-*er1*-8 and KASPar-*er1*-9	*er1*-8, *er1*-9	0.0	VI	Sun et al., 2019
Common root rot (*Aphanomyces euteiches*)	P393/RFLP	*-*		IV	Weeden et al., 2000
	E7M4.251/AFLP, N14.950/RAPD, U326.190/RAPD, E3M3.167/AFLP	*Aph 1, Aph 2, Aph 3*	–	IVb	Pilet Nayel et al., 2002
	E7M4.251/AFLP, U370.900/RAPD, U326.190/RAPD, E3M3.167/AFLP	*Aph 1, Aph 2, Aph 3*	0–2.0	IVb	Pilet Nayel et al., 2005
	AF0164458, AA176, A08_2000, X03_1000, E12_1100	Total 135QTLS most stable QTLS (*Ae-Ps1.2, Ae-Ps2.2, Ae-Ps3.1, Ae-Ps4.1 and Ae-7.6*)	–	I, II, III, IV, V, VI, VII	Hamon et al., 2011
	X03_1000, AB70, A19_800, AF016458, AA430942, E8M2_280, IJB174, J14_850, AB122b	*27 Meta QTLs 2* MQTL-Ae25, MQTL-Ae26	–	I, II, III, IV, V, VII	Hamon et al., 2013
	AA446-486, PA8, AB23-376, AA430942, AB145-364, AD57-300, AA175-282, AB112-402, AD83, AC75-297, PD21-226	Ae-Ps7.6, Ae-Ps4.5, Ae-Ps2.2, Ae-Ps3.1, Ae-Ps5.1	–	II, III, IV, V, VII	Lavaud et al., 2015, Lavaud et al., 2016
	AA122, AA387, AB101	*52 QTLs Major QTLs (Ae-Ps4.4-4.5, Ae-Ps7.6*)	–	IV, VII	Desgroux et al., 2016
	Ps115429/SNP	*Ae-Ps7.6*	–	VII	Desgroux et al., 2018
Ascochyta Blight (*Peyronellaea pinodes)*	p227/RFLP, p105/RFLP, p236/RFLP	QTL	–	IV, II	Dirlewanger et al., 1994
	c206/RFLP, M02-835/RAPD, sM2P5-234/SCAR M27/SCAR, J12-1400/RAPD, C12-680/RAPD, W17-150/RAPD, P346/RFLP, sY16-112/SCAR1 M2P2-193/AFLP sB17-509/SCAR, S15-1330/RAPD	*Asc1.1, Asc2*.*1, Asc3.1, Asc3.2, Asc4.2, Asc4.3, Asc5.1, Asc7.1, Asc7.2, Asc7.3*	–	I, II, III, IV, V, VII	Timmerman-Vaughan et al., 2002, 2004, 2016
	AFLP/RAPD/STS	*ccta2*,*cccc1*, *acct1*	–	II, IV, VI	Taran et al., 2003
	V03-1200/RAPD, PSm PSAA175/SRR, PSMPSAA 163.2/SSR, PSMPSAA399/SSR, G04-950/RAPD, E08-980/RAPD	*mpIII-1, mpIII-3, mpVa-1, mpVII-1, mpVI-1*	–	III, V, VI, VII	Prioul et al., 2004
	DRR230-b, PsDof1	*mpIII-1, mpIII-4*	–	III	Prioul-Gervais et al., 2007
	OPM6598/OPW5387, OPAI141353/OPW21157, OPAI141273/OPAI141353, OPRS4782, OPK6818, OPB111477	*MpIII.1, MpIII.2, MpV.1, MpII.1, MpIII.3, MpIV.1*	–	II, III, IV, V	Fondevilla et al., 2008b
	OPAI14_1353/AA175, OPAI14_1273/OPAI14_1353	Total 14 QTLS, and Major QTLs (*MpIII.3_DRl_06, MpIII.3_DS_06, MpIII.3_DRst_06*)	–	III	Fondevilla et al., 2011
	PsDof1p308/SNP, RGA-G3Ap103/SNP	*-*	–	III, VII	Jha et al., 2015
	PsC8780p118, PsC22609p103, PsC8031p219, PsC20818p367, PsC7497p542, PsC13000p248, PsC4701p407	*abI-IV-1, abI-IV-2, abI-IV-3, abI-IV-4, abIII-1,abVII-1, abI-IV-5, abIII-2, abVII-2*	–	I-IV, III, VII	Jha et al., 2016
	Sc33287_25420/SNP, Sc34405_60551/SNP, Sc33468_44352/SNP, Sc12023_67096/SNP	*abIII-1, abI-IV-2*, abI-IV-2.1, abI-IV-2.2	–	I-IV, III, VII	Jha et al., 2017
	PsC1846p336 - Sc5317_256613/SNP, Sc3030_71736 - PsC7000p195/SNP, Sc8865_149928 - Sc7388_112888/SNP	QTLs	–	IIIb	Gali et al., 2018
	sC8780p118/SNP	*QTL abIII-1*		III	Jha et al., 2019
Ascochyta Blight (*Didymella pinodes*)	OPM4_490/OPK6_887, agpl1_SNP2/MSU515_SNP3, OPZ10_576/Sugtrans_SNP3, sut1_SNP1/OPRS4_699	*MpII.1, MpIII.5, MpV.3, MpV.2*	–	II, III, V	Carrillo et al., 2014b
Pea common Mosaic virus	*p252*	*mo*	15.9	II	Dirlewanger et al., 1994
Pea seed-borne mosaic virus (PSbMV)	GS185/RFLP	*sbm-1*	8.0	II	Timmerman et al., 1993
	G05_2537/RAPD, L01_910/RAPD, P446/RFLP, sG05_2537/STS	*sbm-1*	4.0	II	Frew et al., 2002
Pea enation mosaic virus (PEMV)	CNGC, tRNAMet2	*En*	1.3–2.5	III	Jain et al., 2013
White mold (*Sclerotinia sclerotiorum*)	Chr5LG3_562563492, Chr5LG3_568430003, Chr5LG3_568430003, Chr5LG3_569648908	13 QTLS	–	III	Mahini et al., 2020
Pea weevil (*Bruchus pisorum*)	3546831/DArT, 3551908/DArT, 3548194/DArT, 3552459/DArT, 3549249/DArT, 3549680/DArT,	*BpSI.I*, *BpSI.II* and *BpSI.III, BpLD.I*	–	I, II, IV	Aznar-Fernández et al., 2020
Pea Aphid (*Acyrthosiphon pisum)*	3568590/DArT,3569349/DArT, 3535012/DArT,3536533/DArT, 3535795/DArT, 3537104/DArT, 3568629/DArT, 3536355/DArT	*ApI, ApII, ApIII, ApIV.1, ApIV.2, ApV*	–	I, II, III, IV and V	Barilli et al., 2020
*Pseudomonas syringae* pv. Syringae	OPW5387/RAPD, OPJ121504/OPO61121	*Psy1 and Psy2*	–	III, VI	Fondevilla et al., 2012
Broomrape (*Orobanche crenata*)	STS P48	*Ocp1*	–		Valderrama et al., 2004
	OPM4_978, OPAE5_538, OPP4_479/OPE11_660, OPAA19_702	*n°br03_1, n°br03_2, n°br03_3, n°br04*	–	I, III, V and VI	Fondevilla et al., 2010

## Marker-Assisted Selection

A close association of markers with a trait of interest is the prerequisite of MAS, which identifies the target traits without assessing their phenotype in the early generation (Tayeh et al., 2015a). Both biparental and association mapping approaches have been utilized in the identification of closely associated markers with genes controlling disease resistance in pea. Such gene-linked markers control resistance to PM (Lakshmana Reddy et al., 2015), pea enation or seed borne mosaic virus (Swisher Grimm and Porter, 2020), FW (Jiang, 2013; Kwon et al., 2013), PR (Singh et al., 2015; Barilli et al., 2018), AB (Carrillo et al., 2014b; Jha et al., 2015, 2017), FRR (Coyne et al., 2019), and CRR (Lavaud et al., 2015; Desgroux et al., 2016) and are available for MAB. The marker-assisted backcrossing (MABC) has been successfully used for the introgression of QTLs for *Aphanomyces* root rot (ARR) resistance into several recipient genotypes (Hamon et al., 2013; Lavaud et al., 2015). During the recent years, efforts were made to identify markers closely linked with disease resistance genes. However, such markers are not being widely used in the MAB program for developing resistant cultivars due to their poor linkage with target traits. These efforts have proved the utility of MABC and MAS in pea improvement. Accessibility of the reference genome will pave the way toward finding the genes of interest and understanding the genetic background of individuals at the genome level by deploying molecular markers responsive to high-throughput genotyping.

## Genomics for Understanding the Complex Genetics of Biotic Stress Response and Identification of Candidate Genes

Resistance in the host plant can occur at different stages during compatibility interaction between pathogen and host. Therefore, many mechanisms, metabolic pathways, and proteins are involved in the host plant and pathogen compatibilities. Thus, many genes have to be expressed to control these metabolic pathways or proteins for completing the infectivity of the pathogen with the host plant. Functional knowledge of these genes can help to understand the genetics involved in host plant resistance, which can further be utilized to develop resistant cultivars against a disease. During the recent years, genomic advances have made it possible to know the candidate genes involved in plant resistance by analyzing transcripts of genes expressed during host–pathogen interaction.

### Transcriptomics

Transcriptome analysis has been used to know functional genes responsible for resistance in host plants in many food legumes including pea. In pea, different approaches have been used to recognize the genes responsible for disease and pest resistance (Fondevilla et al., 2011). In the case of white mold [*Sclerotinia sclerotiorum* (Lib.) de Bary], 2,840 host expressed sequence tags (ESTs) (pea) and 996 pathogen ESTs (*S. sclerotiorum*) were identified manifesting exclusively amid the host–pathogen interface, of which about 10% of pea ESTs demonstrated their alliance with genes concerned to its defense against various biotic or abiotic stress, whereas about 9% of *S. sclerotiorum* ESTs exhibited their association with genes reguating pathogenicity or virulence (Zhuang et al., 2012). In another study, microarray analysis investigated gene expression alteration associated with contagion with *D. pinodes* in pea where 346 genes were found to be regulated differentially between resistant and susceptible response, which was responsible mainly for cell wall build-up, phytoalexin and phenylpropanoid metabolism, genes encoding pathogenesis-associated (PR) proteins, and detoxification processes (Fondevilla et al., 2011). The use of deepSuperSAGE identified 17,561 different UniTags, of which about 70% were known sequences from pea or other plants. Among these, 509 UniTags were differentially articulated (Fondevilla et al., 2014). A similar approach was adopted to identify the candidate genes controlling resistance to bacterial blight infection and found a set of about 651 UniTags that expressed differentially between the resistant and susceptible genotypes (Martín-Sanz et al., 2016). In another study, a transcriptome analysis was used to identify the genes and understand the resistance mechanism against *P. pisi* and *A. euteiches* and identified nearly 574 and 817 genes, respectively that were differentially articulated in response to *A. euteiches* contamination at 6 h post-inoculation (hpi) and 20 hpi, respectively, whereas 544 and 611 genes were expressed differentially against *P. pisi* at 6 and 20 hpi, respectively (Hosseini et al., 2015). These genes were associated with phenylpropanoid metabolism, strengthening of the cell wall, and hormonal (jasmonic acid, auxin, and ethylene) signaling (Hosseini et al., 2015). In a comparative transcriptome analysis, contrast responding genotypes to *E. pisi* infection have identified 2,755 transcripts suggesting altered gene expression between the susceptible and resistant genotypes. This study further identified glycolysis as the major pathway of ATP production during pathogen growth and identified genes responsible for putative receptor and regulatory sequences involved in the defense system of resistant genotypes (Bhosle and Makandar, 2021). This information of disease resistant candidate genes can further be utilized for the development of functional markers for MAB.

### Proteomics

Disease and pest infestation trigger changes in the protein profile of the host plant. Knowledge of such protein profiles responsible for compatible interaction between host and pathogen can help in better understanding the host plant resistance mechanism at the molecular level. In addition to this, the abundance of specific proteins can be used as the markers for differentiating resistant and susceptible genotypes, which can be utilized in resistance breeding. Therefore, during the recent years, efforts have been made on proteomic analysis for diseases and pests in pea. Resistance to AB is a complex trait, and infection of this disease alters proteins and their abundance. First protein markers linked to AB resistance have been depicted utilizing resistant and susceptible genotypes. Subsequently, quantitative estimation of these proteins was done in a mapping population for the detection of putative protein markers linked with AB resistance and explored its possible use in breeding (Castillejo et al., 2020). This study eventually developed a group of potential protein markers for resistance to AB and advocated a molecular mechanism against AB resistance in pea. Previously, the proteomic approach identified changes in host proteins during infection of downy mildew in a susceptible cultivar of pea (Amey et al., 2008), of which the levels of eight proteins [PI176 (protein accession number P13239), ABR17 (protein accession number Q06931), glycine-rich RNA-binding protein (protein accession number P49311), cytosolic GAPDH (protein accession number P34922), chloroplastic GAPDH (protein accession number P12858), photosystem I reaction center subunit II (protein accession number Q9S7H1), ATP synthase epsilon chain (protein accession number P05039), and photosystem I iron sulfur center (protein accession number P10793)] increased significantly in the infected leaves of the susceptible plant. Identification of these proteins provided the base for the advancement to reveal molecular defense mechanisms to *P. viciae* infection (Amey et al., 2008). In another study, proteomic analysis of PM susceptible and resistant genotypes resulted in the identification of proteins concerned with photosynthetic activity and carbon metabolism, signal transduction functions, protein synthesis, and protein degradation, which aids in understanding the mechanisms of *E. pisi* resistance in pea (Curto et al., 2006). Similarly, in a recent study, proteomic analysis was done for PM isolates infecting susceptible pea cultivar and identified proteins involved in virulence and pathogenesis through signal transduction, secondary metabolite formation, and stress functions (Bheri et al., 2019). For understanding the resistance mechanism to *Acyrthosiphon pisum* (pea aphid), a serious pest of pea, proteomic analysis between contrasting genotypes identified the proteins mostly corresponding to amino acid metabolism, carbohydrate metabolism, folding or degradation, stress response, photosynthesis, signal transduction, and transcription or translation suggesting the role of different metabolic pathways in controlling resistance to this pest (Carrillo et al., 2014a). Thus, proteomic analysis has provided better insight into the molecular mechanism underlying disease and pest resistance in pea, and hence, it is further required to enhance the understanding of the molecular mechanism of quantitatively inherited diseases and pests resistance in pea.

## Future Breeding Strategies for Developing Cultivars Resistant to Biotic Stresses

### Development of Functional Markers

Poor association of molecular markers with genes/QTLs controlling disease resistance has led to their limited use for MAS in pea breeding programs. Therefore, the development of the functional markers within targeted genes/QTLs controlling the disease resistance is important for this purpose. Earlier, few efforts have been made to develop functional markers for the *er1* gene controlling PM in pea (Sun et al., 2016, 2019). A functional co-dominant CAPS marker with 94% accuracy was found useful for the selection of resistance genes responsible for *F. oxysporum* race 1 (Jain et al., 2015). Furthermore, next-generation sequencing also assisted in developing functional SNP markers from genes/QTLs governing resistance to different diseases in pea. For example, SNP markers within two candidate genes (*PsDof1* and *RGA-G3A*) were identified for AB resistance (Jha et al., 2015). Association mapping with a large number of SNP markers developed through next-generation sequencing identified SNP marker, associated with a major QTL *Ae-Ps7.6* responsible for reducing ARR severity and root system architecture (RSA). Therefore, the identified genes for RSA could be utilized in improving ARR incidence in pea. Furthermore, the availability of a reference genome sequence of pea along with a high-throughput next-generation genotyping platform provides the opportunity to identify the candidate genes for targeted traits and development of functional markers linked with disease resistance genes for marker-assisted breeding in pea.

### Toward Genomic Selection in Pea

For obtaining maximum genetic gain with more accuracy, genomic selection (GS) using molecular markers is a promising approach. This can help to improve biotic stress resistance, which is a primary breeding objective of the pea genetic improvement program. This approach is more useful for improving quantitatively inherited disease resistance in pea. It uses genome-wide molecular markers associated with resistance genes for predicting and selecting high breeding value lines. In a recent review, different models used in GS were discussed in detail; particularly, the use of multivariate GS models (MTGS) over single trait GS (STGS) was presented (Budhlakoti et al., 2019). Multi-trait GS (MTGS) methods may provide more accurate genomic-estimated breeding values (GEBVs). Several MTGS methods were used for GS, e.g., the multivariate mixed model approach (Jia and Jannink, 2012; Klápšě et al., 2020), Bayesian multi-trait model (Jia and Jannink, 2012; Cheng et al., 2018), multivariate regression with covariance estimation (MRCE) (Rothman et al., 2010), and conditional Gaussian graphical model (cGGM) (Chiquet et al., 2017). Jia and Jannink (2012) presented three multivariate linear models (i.e., GBLUP, Bayes A, and Bayes Cπ) and compared them with univariate models. Most of the successful events of the utilization of GS in biotic stress resistance were in cereal crops. In wheat, GS was used for three types of rust, Fusarium head blight, septoria tritici blotch, PMD, tan spot, and *Stagonospora nodorum* blotch (Budhlakoti et al., 2022). The genomic prediction accuracies for these diseases ranged from 0.14 to 0.85 (Daetwyler et al., 2010; Rutkoski et al., 2012; Mirdita et al., 2015; Juliana et al., 2019; Sarinelli et al., 2019). Similarly, in the case of rice, GS has been used in blast disease tolerance (Huang et al., 2019). In maize, GS has been used against *Stenocarpella maydis* causing ear rot (Dos Santos et al., 2016) and heavy infestation of Striga (Badu-Apraku et al., 2019). In the case of barley, for Fusarium head blight, the prediction accuracy was 0.72 (Lorenz et al., 2012; Sallam and Smith, 2016). Though limited reports of the use of genomic selection to improve biotic stresses in pea are available, efforts have been made to know the impact of the marker density, statistical method, and/or the training population size for evaluating genomic prediction accuracy using the number of seeds per plant, thousand seed weight, and flowering time. Such information provides opportunities for developing GS strategies (Tayeh et al., 2015b), which is important for biotic stress tolerance in pea.

### Mining Allelic Variants for Resistance Genes

Breeding for improving a trait requires ample availability of diversity in germplasm for the targeted traits. In pea, a large collection of genetic resources is available, which are a reservoir of undiscovered allelic variants for many traits (Tanksley and McCouch, 1997; Smýkal et al., 2012). This large collection may have new resistant allele(s) of the gene(s) controlling disease incidence in pea. For mining such alleles from germplasm, there is a need to test the entire germplasm for their response following a specific screening protocol, which is not only time-consuming but also expensive. However, current genomic tools have provided an opportunity to uncover the allelic variation, especially for those monogenic traits for which candidate genes are already known (Robaglia and Caranta, 2006; Hofinger et al., 2011; Reeves et al., 2012). The use of such genomic tools increases the identification of allelic variants for resistance genes by screening the wild and cultivated germplasm in several crops (Bhullar et al., 2009). In pea, eukaryotic translation initiation factor 4E provides resistance against many potyviruses. Therefore, gene *eIF4E* encoding this factor has been used for the identification of allelic diversity among 2,803 pea accessions, which resulted in the identification of four *eIF4EA-B-C-S* variants, whose distribution was geographically linked, suggesting its independent evolution (Konečná et al., 2014). This study has opened an avenue of research for the identification of new allelic variants for complex diseases of a pea.

### Toward Epigenetic Breeding

Transgenerational epigenetic variation, which transfers steadily to the next generation, becomes one of the important strategies for breeding climate-resilient cultivars in crop plants. These variations cause alteration in gene expression through DNA methylation or histone modification (Kumar et al., 2019). Identification or genome-wide mapping of epigenetic markers can help the breeder to manipulate epigenomic variability toward the development of climate resilient crop varieties. This epigenetic variation was detected in host plant resistance against a broad array of plant pathogens such as fungi, bacteria, viruses, nematodes, oomycetes, and herbivorous insects (Espinas et al., 2016; Ramirez-Prado et al., 2018; Alonso et al., 2019). For example, in soybean, methylome has been identified for compatible interaction of roots with cyst nematodes (Rambani et al., 2015). In pea, differences have been detected for methylations among plants, which were propagated through *in vitro* culture for a long time (Smýkal et al., 2007). Artificially induced and naturally occurring epigenetic variations controlling plant disease resistance were identified, and similar efforts are required to identify epigenetic variation responsible for polygenetically inherited disease resistance in pea. In pea, no potential genetic sources for resistance are available so far for many serious diseases, and hence, new epigenetic alleles can be generated using promising approaches such as induced gene-specific DNA methylation and epigenome editing (Zhi and Chang, 2021). Thus, epigenetic breeding has a great potential for improving disease resistance in pea.

### Genome Editing

In pea, insect pests and diseases are the major yield-limiting factors and hence pose a substantial threat to food security globally. In recent years, genome editing or modification has revolutionized the functional analyses of genes and the introduction of new alleles for the trait of interest into commercial crop plants (Mushtaq et al., 2019). Different approaches of genome editing have been developed for this purpose; however, clustered regularly interspaced short palindromic repeats (CRISPR)/CRISPR associated protein 9 (CRISPR-Cas9), meganucleases, transcription activator-like effector nucleases (TALENs), and zinc-finger nucleases (ZNFs) are being used extensively for genetic improvement (Mushtaq et al., 2019). In crop plants, susceptibility (*S*) or resistance (*R*) genes have been considered eventual targets intended for escalating crop protection (Singh et al., 2016; Ren et al., 2017). These genes were identified as the best candidate for gene editing for conferring disease or pest resistance in a crop (Das et al., 2019b). In addition to this, editing of most conserved regions of multiple viral genomes using multiplex CRISPR/Cas9 system also helped in conferring disease resistance in various crops by interfering with their duplication and progress (Iqbal et al., 2016). In pea, the transcriptomic analysis provides elucidation of the genes and pathways concerned with disease or pest resistance. Moreover, the study of expression alteration, modification, and interaction of protein during the plant-pathogen interface provided knowledge of key proteins involved in pathogenesis. This information is a useful repository for editing or modification of the genome of a crop or realtered pathogen toward the development of resistant cultivars (Barakate and Stephens, 2016). In addition to this, genome editing can be used to alter epi-alleles or to generate new epi-alleles involved in disease resistance (Latutrie et al., 2019).

### Transgenic Technology

In pea, limited resistance sources are available among cross-compatible germplasm for several devastating diseases and insect pests such as FRR, CRR, PR, alfalfa mosaic virus, and bruchids. Therefore, transferring resistance genes from other non-cross-compatible species is one of the ways to develop resistant cultivars, possibly by developing transgenic plants. However, genetic transformation in pea is not easy when compared to other legume crops due to difficulties in transformation and plant regeneration (Svabova et al., 2005; Warkentin et al., 2015). Although, during the recent years, advances in biotechnology have made possible the development of transgenics in pea for diseases and insect pests. For example, transgenic lines with two chimeric genes encoding the coat protein (CP) of alfalfa mosaic virus (AMV) strain NZ1 have been developed and tested under green house and field conditions for improved AMV resistance in pea. However, results showed partial virus resistance of transgenic lines having genetically modified AMV CP sequences (Timmerman-Vaughan et al., 2001). In another study, two antifungal genes (chitinase and glucanase) for resistance to fungal diseases have been transferred using genetic transformation, and transgenic pea has been developed by stacking these genes (Amian et al., 2011). Weevils are the most devastating insect of food legumes including pea. Genetic resistance to this insect is not available currently in cross-compatible germplasm. However, a gene for alpha-amylase inhibitor-1 (αAI) has been identified in the common bean that completely protects from weevil destruction. This has been transferred through a genetic transformation in pea, and developed transgenic lines showed resistance to this pest. Moreover, αAI transgenic peas are found to be less allergenic than beans or non-transgenic peas in mice (Reiner et al., 2013).

In a more recent study, four antifungal genes, *1-3 β glucanase* (G), *endochitinase* (C) (belonging to the PR proteins family), *polygalacturonase inhibiting proteins* (PGIPs) (P), and *stilbene synthase* (V), have been transformed for disease tolerance in European pea cultivars. This resulted in the development of transgenic lines having an individual antifungal gene or all four genes that were stacked through hybridization. However, the resistance of these transgenic lines against FRR was not consistent over the years in confined field trials probably due to lower relative gene expression in the roots (Kahlon et al., 2018). Although, these studies showed the possibility of developing transgenic pea against major diseases and insect pests. Thus, transgenic technologies have great promise but the economic benefits of genetically modified (GM) pea will need to surpass the regulatory costs, time, and labor involved in bringing a GM crop to market. In addition to this, more research experiments are required on issues associated with genetically modified crops, such as discrete changes in the molecular architecture, cellular function, and antigenicity of the expressed protein translated from the transferred gene in the transgenic plants. In pea, transgenic expression of a plant protein (alpha-amylase inhibitor-1) from the common bean, which is a non-native host of pea, led to the synthesis of a structurally modified form of this inhibitor. The effect of this modified protein has been studied in mice and found that non-native proteins in transgenic plants may lead to structural modification with altered immunogenicity (Prescott et al., 2005).

### Speed Breeding

Environmental conditions play an instrumental role in making crop plants susceptible to biotic stresses. The changing environmental condition due to global warming provides opportunities for evolving new races and pathogens, which has significantly raised concern for meeting global food security. Therefore, there is an urgent need of developing resistant cultivars within a short period of time. However, present breeding approaches take several years to develop the resistant cultivars, and hence, the current improvement rate is inadequate to meet the future food demands. Elongated generation advancement time of crops is one of the key reasons for delay in the development of improved resistant cultivars against biotic stresses. Therefore, in recent years, speed breeding has emerged as a powerful tool for accelerating crop research and breeding as several workers have developed speed breeding protocols in pea for shortening the breeding time (Ghosh et al., 2018; Watson et al., 2018; Cazzola et al., 2020). These speed breeding techniques along with new biotechnological tools available in pea can accelerate the development of resistant cultivars against new emerging pathogens or races due to climate changes in the following way:

• Taking 4–5 breeding generations in a year could substantially reduce the time span to release a variety.
• Development of RIL mapping populations within a short period of time using speed breeding can help in the rapid identification of QTLs for disease resistance and their use in the breeding program for developing improved resistant cultivars.
• The MABC for introgression of QTLs/genes controlling disease resistance can be faster through speed breeding leading to the rapid development of improved and resistant cultivars.
• The amalgamation of speed breeding with other modern breeding and biotechnological techniques such as genome editing, genomic selection, and high-throughput genotyping has great potential for accelerating the genetic gain toward the development of biotic stress-tolerant cultivars.

## Conclusion and Perspectives

**FIGURE 2:**
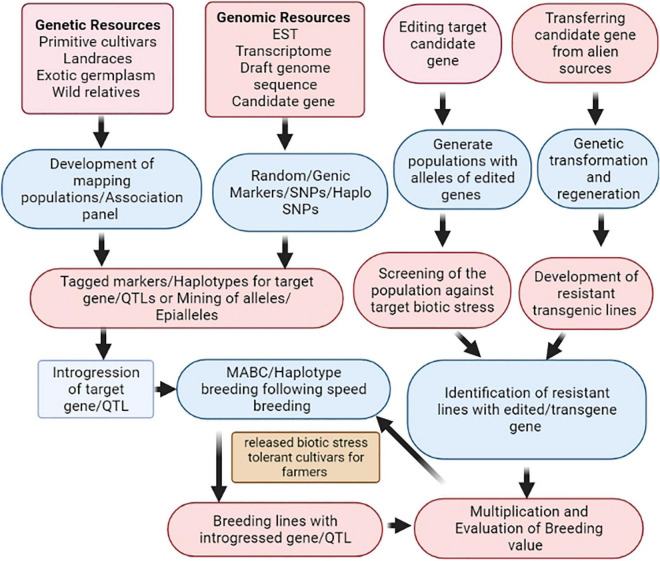
Genomic-assisted breeding strategies for biotic stress tolerance.

Pea is an important and exceptionally high-yielding cool season pulse crop in the world. Numerous biotic stresses are the key constraints in harnessing the full production potential of a pea, of which fungal diseases such as PM, FW, FRR, AB, CRR, and PR causing infection during different growth stages are devastating to the crop. Nevertheless, sincere efforts have been made to elevate the productivity and production of pea, but many more milestones are yet to be achieved for making it a resilient crop to upcoming challenges. Several major and minor genes/QTLs governing important biotic stresses in pea have been dissected and mapped using existing genomic tools, nevertheless, not utilized to a large extent in regular pea breeding programs. The reliable DNA markers flanking the genes/QTLs of interest could accelerate the introgression of resistance from the resistance sources using the genomic-assisted protocol to speed up the pea breeding program accomplishments more efficiently and precisely. Updated research efforts are warranted for the amalgamation of next-generation genomics and phenomics in pea improvement programs. The schematic diagram explains how different genomic approaches can be combined to accelerate the success of a pea breeding program (Figure 2). This figure also explains the combined use of genetic resources, genomic resources, and advanced biotechnological tools in the pea improvement program for the development of biotic stress-resistant cultivars. Underlying resistance mechanisms for AB, PM, and pea aphids have been elucidated using different pathogenic resistance proteins pertinent to the genes and pathways involved in pathogen resistance. However, more concentrated efforts are needed in the future on proteomic and transcriptomic analyses to untangle the disease and pest resistance mechanism in pea at the molecular level and to validate the sequencing results at the functional level for the identification of candidate genes controlling biotic stress resistance. This information will be certainly useful for editing or modification of crop genomes or realtered pathogens to develop resistant cultivars. Genome-wide association and genomic selection, which elucidate specific genetic variations at the genome scale, should be judiciously used for the identification of several gene(s)/QTLs exerting smaller effects on the biotic stress resistance. The transgenic technology should be exploited to let researchers utilize the variability existing outside the crop’s primary/secondary gene pool and also offer an opportunity to conquer crossability constraints. In addition, induced gene-specific DNA methylation and epigenome editing can be exploited to generate new epigenetic alleles for different biotic stresses. Most recently, speed breeding or rapid generation advancement protocols developed for shortening breeding times (4–5 cycles/year) have emerged as a potent technology for accelerating genetic gain in pea. Though, several tools and technologies are in hand judicious use to reap the best of them is challenging, certainly, there is a huge scope to achieve new heights in productivity enhancement by breeding biotic stress-resistant pea cultivars.

## Author Contributions

All authors listed have made a substantial, direct, and intellectual contribution to the work, and approved it for publication.

## Conflict of Interest

The authors declare that the research was conducted in the absence of any commercial or financial relationships that could be construed as a potential conflict of interest.

## Publisher’s Note

All claims expressed in this article are solely those of the authors and do not necessarily represent those of their affiliated organizations, or those of the publisher, the editors and the reviewers. Any product that may be evaluated in this article, or claim that may be made by its manufacturer, is not guaranteed or endorsed by the publisher.
